# Bariatric surgery exclusion: Psychiatric causes

**DOI:** 10.1192/j.eurpsy.2021.1867

**Published:** 2021-08-13

**Authors:** C. Vilella Martín, P. García Vázquez, I. Gonzalez Rodríguez, S. Nuñez Sevillano, A. Serrano García

**Affiliations:** Psychiatry, Complejo Asistencial Universitario de León, León, Spain

**Keywords:** eating disorders, impulse control disorder, Bulimia Nervosa, Bariatric surgery

## Abstract

**Introduction:**

The psychopathological causes that advise against a bariatric surgical procedure include any state that puts at risk the modification of habits and beliefs regarding eating behavior, wich condition weight loss and health improvement.

**Objectives:**

To Study the psychiatric profile of patients rejected for bariatric surgery at the Complejo Hospitalario Asistencial de León (León, Spain).

**Methods:**

Retrospective observational study. All patients for whom bariatric surgery procedure has been contraindicated for psychopathological reasons are included. 145 patients were evaluated in the context of the protocol for bariatric surgery. The following diagnostic scales were used as support: Salamanca Questionnaire, Plutchik Impulsivity Scale, Attitudes towards change in patients with eating disorders (ACTA), Bulimia Investigatory Test Edinburgh e, and European Quality of Life-5 Dimensions.

**Results:**

41 Patients were rejected for psychiatric reasons (28.28%). The most frequent diagnoses are impulse control disorder (39%), followed by eating disorder (27%). Other diagnoses found are: depressive disorder (10%), adjustment disorder (5%), personality disorders, intellectual disability and generalized anxiety disorder (3%) 78% of them are women.

**Conclusions:**

Uncontrolled psychiatric pathology is a contraindication to bariatric surgery. Impulse control disorder and eating disorder are related to overweight and obesity, so a diagnosis and treatment are necessary prior planning surgical procedure. Psychopathological variables determine the success of bariatric surgery procedures and it is mandatory to consider them in the process.

**Disclosure:**

No significant relationships.

